# Genomics Reveal Staphylococcus aureus Persists during Long-term Urinary Catheterization Despite Antimicrobial Therapy and Catheter Exchanges

**DOI:** 10.21203/rs.3.rs-6702271/v1

**Published:** 2025-05-29

**Authors:** Jennifer Walker, Jesus Duran Ramirez, Chelsie Armbruster, Blake Hanson

**Affiliations:** The University of Texas Health Science Center; The University of Texas Health Science Center; University at Buffalo, State University of New York; University of Texas Health Science Center at Houston

**Keywords:** CAUTI, asymptomatic bacteriuria, Staphylococcus aureus persistence, long-term human urinary catheterization

## Abstract

Urinary catheters, the most frequently placed medical devices in the US, increase the risk of developing symptomatic catheter-associated urinary tract infection (CAUTI) and asymptomatic bacteriuria (ASB) – the presence of bacteria in the urine – with those requiring long-term urinary catheters (LTUCs) at highest risk. While ASB and CAUTI are caused by a broad range of uropathogens, most remain understudied. We used whole-genome sequencing to investigate the understudied uropathogen, *Staphylococcus aureus.* Analysis of 153 longitudinal *S. aureus* isolates previously collected from urinary catheter or urine samples from 20 individuals with LTUCs (average of 8 longitudinal isolates/person) demonstrated that most strains were multidrug resistant (MDR), including methicillin-resistant *S. aureus* (MRSA), and sequence type 5. Importantly, the same *S. aureus* strain persisted as ASB for an average of 13 weeks, despite antibiotic exposures or urinary catheter exchanges, and in one case transitioned to symptomatic CAUTI. The longitudinal strains were highly genetically related with few genomic changes and stable antimicrobial resistance and virulence gene carriage. This work demonstrates that MDR *S. aureus* can persist as ASB long-term and that common strategies to reduce or eliminate microbes from LTUCs were ineffective at eradicating the uropathogen in most cases, despite phenotypic susceptibility to the administered antibiotic.

## Introduction

Urinary catheters are the most frequently placed medical devices in the United States, with > 30 million used annually^1,2^. Their widespread use is due to their efficacy in the clinical management of hospitalized and critically ill patients, of which 25% and 60% receive a urinary catheter, respectively^3^. Urinary catheters are also extremely effective at improving the quality of life for millions of individuals, including those with neurogenic bladder and the elderly^4,5^. However, urinary catheters increase the risk of developing symptomatic catheter-associated urinary tract infections (CAUTIs) – with individuals requiring long-term (> 28 days) urinary catheters (LTUCs) at highest risk^6,7^. Additionally, urinary catheters increase the incidence of asymptomatic bacteriuria (ASB) – the presence of bacteria in the urine without symptoms^3^. Persistent ASB, particularly among those with LTUCs, also increases the risk of developing symptomatic CAUTI^8,9^. Challengingly, studies demonstrate that prophylactic antibiotic treatment of individuals with urinary catheters does not eliminate ASB nor does it prevent catheter colonization, even during short-term (< 28 days) catheterization^3,10-12^. Prophylactic antibiotic treatment is, however, associated with the development of antimicrobial resistance (AMR), as well as other serious illnesses, including *Clostridioides difficile* colitis^13,14^. Additional studies indicate empiric antibiotic treatment of CAUTI also does not improve patient outcomes^15,16^. Thus, management of ASB and prevention and treatment of CAUTI remain difficult.

Due to the high infection rates of urinary catheters (10–30%) combined with a lack of prevention strategies, CAUTIs have become the most common hospital-associated infections in the U.S., accounting for > 1 million cases and resulting in as much as $1.83 billion/year in associated preventable costs ^17-19^. CAUTIs can also progress to severe disease, including bacteremia and sepsis, which increases healthcare costs, length of hospital stay, morbidity, and mortality ^20-22^. Both CAUTI and ASB are caused by a broad range of uropathogens, including Gram-negatives such as *Escherichia coli, Klebsiella pneumoniae,* and *Pseudomonas aeruginosa,* as well as Gram-positives like *Enterococcus faecalis* and *Staphylococcus aureus* ^23,24^. Increasing AMR among these uropathogens not only limits treatment options, but it further complicates empiric treatment ^25,26^. Additionally, CAUTI and ASB in individuals with LTUCs are typically polymicrobial, making it difficult for physicians to differentiate uropathogens that cause symptoms from those that are asymptomatically colonizing ^27,28^. Despite the significant challenges associated with identification and treatment of these diverse uropathogens, the majority remain understudied. Understanding how these uropathogens persist as ASB despite prophylactic antibiotic administration is critical for developing and implementing treatment strategies that reduce the disease burden associated with these common and costly infections.

In this study, we focus on the understudied uropathogen *S. aureus.* Previous studies demonstrate that *i) S. aureus* within the urinary tract is primarily associated with urinary catheterization, *ii)* for those with persistent *S. aureus* ASB, one third will go on to develop a symptomatic UTI, and *iii)* UTI symptoms caused by *S. aureus* are typically more severe, with higher rates of dissemination to secondary bacteremia, compared to other uropathogens^9,29-33^. Additionally, recent studies indicate *S. aureus* is a common asymptomatic colonizer of LTUCs; yet there is a paucity of studies investigating the lifecycle of this pathogen in the urinary tract, particularly within LTUCs^3,11^. We used whole-genome sequencing to analyze a collection of *S. aureus* strains previously isolated from 20 individuals with LTUCs^3,11^. A total of 171 strains, with an average of 8 longitudinal isolates collected per person over an average collection duration of 9 months, were included in these analyses. We discovered that most individuals were colonized with methicillin resistant *S. aureus* (MRSA) belonging to multi-locus sequence type (ST) 5. Notably, most participants received multiple antibiotics over the course of the collection period, with nitrofurantoin and ciprofloxacin the two most commonly prescribed^3,11^. Antimicrobial susceptibility testing revealed all *S. aureus* isolates tested were susceptible to nitrofurantoin, but > 60% were resistant to ciprofloxacin. Despite anti-staphylococcal antibiotic exposure and frequent catheter exchanges, longitudinal isolates from a given participant – termed within-participant – were highly genetically related, with a median of four changes (gene gain/loss) to the accessory genomes and a median pairwise SNP distance of seven single nucleotide polymorphisms (SNPs). Virulence factor and AMR determinant carriage was also stable among within-participant isolates. Furthermore, two participants developed symptomatic *S. aureus* CAUTI during the collection period, of which one of the *S. aureus* isolates was detected as ASB for months prior to and after the symptomatic events. These results suggest that the two strategies used most frequently to try to reduce or eliminate ASB (antimicrobial administration^34^ and catheter exchange^7^) are ineffective at preventing *S. aureus* from persisting in individuals with LTUCs. Collectively, this study demonstrates *S. aureus* persistently colonizes the catheterized urinary tract, serving as a reservoir for AMR makers and subsequent symptomatic infection.

## Results

### Epidemiology of S. aureus isolates from individuals with LTUCs

*S. aureus* strains were longitudinally isolated from individuals with LTUCs from two separate, previously published cohorts^3,11,12^. The first cohort consists of urology outpatients (UPs) from a single outpatient clinic^11,12^. For this cohort, microbes, including *S. aureus,* were cultured monthly from urine and LTUCs for approximately 12 sampling time points. The second cohort consists of nursing home residents (NHRs) from two long-term care facilities^3^. For this cohort, microbes, including *S. aureus,* were cultured weekly from urine samples for up to 27 sampling time points. While the sample collection type and sites of each cohort differed, the average participant age was similar, 63.4 (± 15.5 years) for the UP and 65 (±15 years) for the NHR cohorts^3,11^. Additionally, each cohort consisted of 10 participants that were colonized with *S. aureus* during at least one sampling time point (Table 1). For the 10 UPs, they received an average of 2.9 antibiotic treatments per participant, 50% were female, and *S. aureus* strains were collected on average every 27.4 days at the time of the scheduled catheter exchange. For the 10 NHRs, they received an average of 1.5 antibiotic treatments per participant, 20% were female, and *S. aureus* strains were collected on average every seven days – with catheter exchanges occurring every 38.12 days on average. There were no significant differences observed for the gender, antibiotic exposures, or catheter duration between the UPs and NHRs colonized with *S. aureus.* Overall, the demographics and epidemiology of individuals with *S. aureus* colonization within both cohorts were similar.

### S. aureus LTUC isolate collection

**Figure 1:**
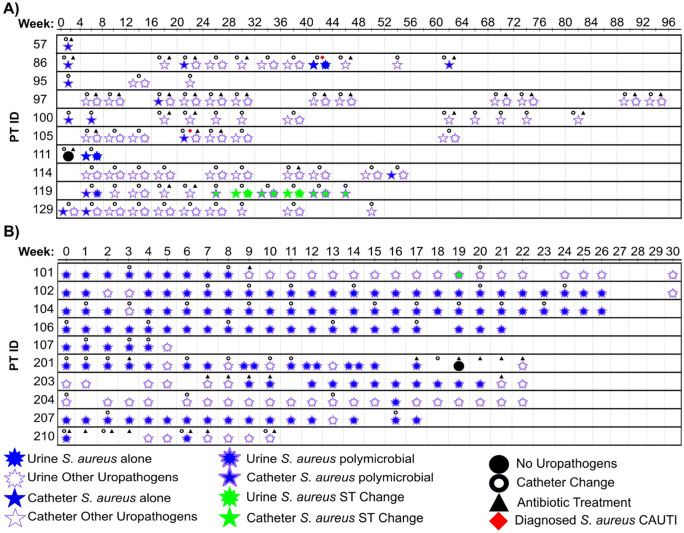
Sample collection period of *S. aureus* mono- or poly-microbial urinary tract colonization, antimicrobial exposures, diagnosed *S. aureus* catheter-associated urinary tract infection (CAUTI), and catheter exchanges. **A)** The urology outpatient (UP) isolates were collected from catheter and urine samples during regularly scheduled, monthly catheter exchanges. A total of 38 longitudinal *S. aureus* isolates were collected and two individuals experienced symptomatic *S. aureus* CAUTI. **B)** The nursing home resident (NHR) isolates were collected from weekly urine samples. A total of 133 *S. aureus* strains were collected and no individual experienced a symptomatic *S. aureus* CAUTI.

A total of 171 *S. aureus* isolates were collected from 273 sampling time points across these 20 participants (**Table S1**). Most participants had a single isolate per species collected at each time point. However, several individuals had multiple *S. aureus* strains collected within a single time point – defined as co-isolated. These co-isolated strains were collected based on phenotypic differences, including size, color, rugosity, and antibiotic resistance, and were more frequent in the UP cohort due to culturing of both urine and catheter samples^3,11^. Whole-genome sequencing confirmed all 171 strains were *S. aureus* (**Table S1-S3**). Within the UPs, 38 *S. aureus* isolates were sequenced, averaging 3.8 strains per participant (Fig. 1, Tables 1 and S1-S3). Within the NHRs, 133 strains were sequenced, averaging 13.3 isolates per participant (Fig. 1, Tables 1 and S1-S3). Notably, two participants within the UP cohort experienced symptomatic *S. aureus* CAUTI^11^. None of the participants in the NHR cohort were diagnosed with CAUTI attributed to *S. aureus*^3^. While the majority of samples collected from these 20 participants were polymicrobial, *S. aureus* was observed to be monomicrobial at four separate time points (Fig. 1). Despite multiple antibiotic exposures and catheter exchanges over the course of sample collection, only two of the 273 samples collected had no detectable uropathogens across both cohorts. These results indicate that *S. aureus* is a common, asymptomatic colonizer that can be mono-microbial within individuals with LTUCs.

### Genomic analyses of S. aureus LTUC co-isolated strains

To determine the population heterogeneity of co-isolated strains and investigate whether strains isolated from the urine and LTUC were genetically related, we analyzed 29 co-isolated genomes from four patients across 10 time points (Fig. 1 **and Table S1**): NHR 201 at collection 9 (n = 2; both urine), 12 (n = 2; both urine), and 14 (n = 2; both urine); UP 86 at collection 7 (n = 2; 1 catheter, 1 urine) and 10 (n = 2; both catheter); UP 111 at collection 0 (n = 2; 1 catheter, 1 urine); and UP 119 at collection 0 (n = 2; 1 catheter, 1 urine), 7 (n = 9; 6 catheter, 3 urine), 8 (n = 4; 1 catheter, 3 urine), and 10 (n = 2; 1 catheter, 1 urine). Strains isolated from the same collection time point were the same ST (**Table S2**). Additionally, analyses of the accessory genome and SNPs (**Table S4-5**) revealed that co-isolates displayed a median of 2.5 (IQR 1-12.5) accessory genome changes and a median pairwise SNP distance of five (IQR 3–7), regardless of culture site or colony phenotype (**Figure S1A** and **S1B**). Notably, both MRSA and methicillin-susceptible *S. aureus* (MSSA) were co-isolated from NHR 201. In-depth analyses of genomic differences between the MRSA and MSSA co-isolates (n = 2; two collection times) revealed that despite both lineages sharing the same ST (ST5), there was a median of 72 (IQR 69–75) accessory genomes changes and a median pairwise SNP distance of 50 (**Figure S1C** and **S1D**). These results suggest these co-isolates are from different lineages, despite isolation at the same collection time and sharing the same ST. Together, these findings indicate that most participants had a single, homogenous population of *S. aureus* with few genomic differences that predominated at each collection time, and that the strain colonizing the catheter is highly genetically related to the strain colonizing the urine.

### Phylogenetic analyses of longitudinal S. aureus LTUC isolates

**Figure 2:**
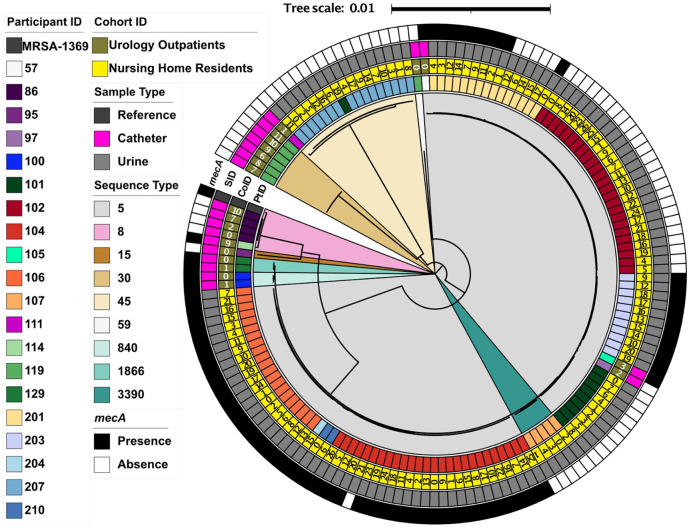
Phylogenetic analysis of longitudinal *S. aureus* isolates collected from individuals with long-term urinary catheters. The phylogenetic tree displays the participant ID (PT ID), the cohort ID and collection time point (CO ID), sample type (SID), and the presence or absence of *mecA* (*mecA*) moving from the inner to the outer rings, and the multi-locus sequence type (ST) within the branches of the tree. Isolates cluster first by ST type, and then within each ST by participant.

To determine whether longitudinally collected *S. aureus* LTUC isolates among both cohorts undergo frequent strain replacement – due to antibiotic exposure and catheter exchanges – or if they are the same strain persisting over time, a total of 153 longitudinal strains were selected for further analyses, representing a single strain from each participant at each collection time. Approximately 40% of UP and 60% of NHR strains carried *mecA,* classifying them as MRSA (Table 1 and Fig. 2). The most common lineage was ST5, which colonized 55% (11/20) of participants (Figs. 2, S2, and S3). The second most common lineage was ST45, which colonized 15% (3/20) of participants, followed by ST8 found in 10% (2/20) of participants. Impactfully, most participants (90%; 18/20) were colonized with the same ST over time (Figs. 1 and 2). There were only two instances (a single participant from each group) where the initial ST detected changed to a different ST at a later collection time: NHR 101 and UP 119. Phylogenetic analyses, using the well-characterized *S. aureus* strain MRSA-1369 to root the tree, demonstrated that within-participant longitudinal isolates of the same ST clustered together (Figs. 2, S2, and S3). The longitudinal isolates with different STs – from the participants that experienced the ST changes – clustered separately. Additionally, participant isolates were observed to form sub-clusters based upon *mecA* carriage (Figs. 2 and S3). Specifically, the longitudinal MSSA and MRSA isolates colonizing NHR 201 at alternating time points across collection times each formed distinct clusters, separate from one another. Together, these clustering patterns suggest that within-participant, longitudinal *S. aureus* isolates are more closely related to each other than they are to isolates from other participants, even from the same ST. Importantly, this pattern was observed across geographically distinct cohorts, suggesting these results are broadly applicable to individuals with LTUCs colonized with *S. aureus.*

### Accessory genome analyses of longitudinal S. aureus LTUC isolates

**Figure 3:**
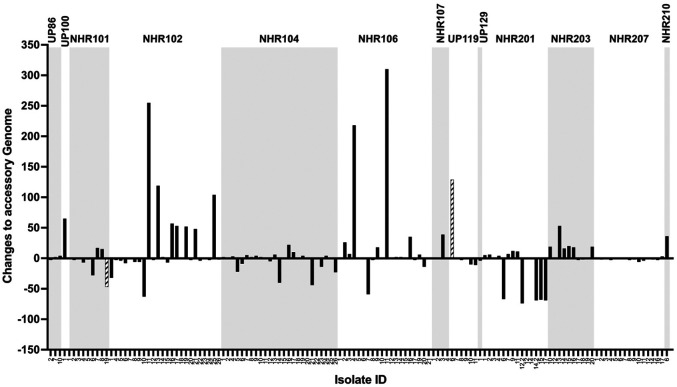
Changes in accessory genes over time. Changes in the accessory genomes are shown as changes from the index isolates: a positive number indicates a gain of genes, a negative number denotes a loss of genes, a value of 0 represents no change. Black bars represent comparisons of longitudinal isolates with the same ST while hatched bars represent strains with a new ST (strain switching). Most within-participant isolates maintained a similar accessory genome over time.

To determine whether there were changes in the gene repertoire of longitudinal *S. aureus* isolates over time, a pan-genome analysis was performed (**Table S4 and Figure S4**). Changes to the accessory genome were determined by comparing the initial isolate to each subsequent longitudinal isolate. For within-participant longitudinal isolates with STs that differed from the index isolate (UP 119 and NHR 101), a median of 126 (IQR 118–129) accessory genome changes were observed (Fig. 3). In contrast, comparisons between the first strain isolated of the same ST as the subsequent longitudinal isolates from UP 119 and NHR 101 revealed a median of 3 (IQR 1–13) accessory genome changes. Thus, the first occurrence of a new ST was determined to be an index isolate for all subsequent longitudinal isolates that were the same ST. Importantly, comparisons of all longitudinal isolates to their respective index isolate demonstrated that a median of four (IQR 1–19) accessory genome changes were observed (Fig. 3). Additionally, the NHR 201 index (MRSA) isolate compared to the five longitudinal MSSA strains indicate a median of 69 (IQR 67.50–71.50) genes are absent from the MSSA genomes (Fig. 3 and S5A). Most absent genes were associated with the SCC*mecA* Type II (2A) cassette, which is present in the MRSA isolates. Notably, comparing the first MSSA strain isolated (NHR 201-6) to the longitudinal MSSA strains, a median of 3 (IQR 1-6.5) accessory genome changes were observed (**Figure S5B**). Together, these findings indicate that there are few changes to the accessory genomes of longitudinal isolates with the same ST and *mecA* carriage over time.

### SNP analyses of longitudinal S. aureus LTUC isolates

**Figure 4:**
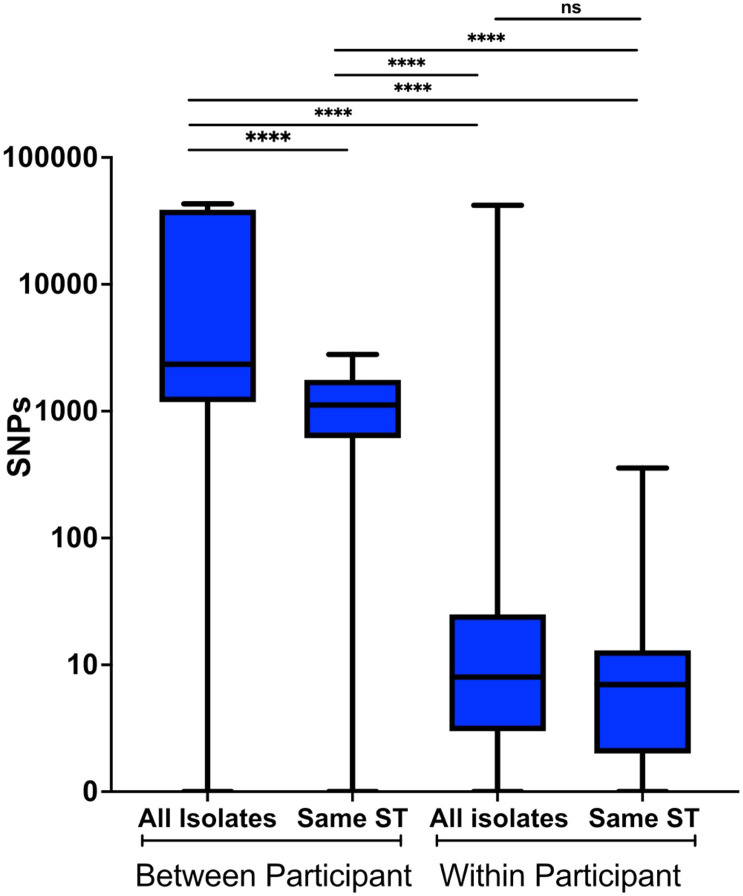
Single nucleotide polymorphism (SNP) analysis. Comparisons include all isolates between participants (n=3344, median = 2344), all isolates of the same sequence type (ST) between participants (n=1302, median =1120), all isolates within a participant (n=148, median = 8), and isolates of the same ST within a participant (n=131, median = 7). Box and whisker plots display the median as a black bar within the interquartile range (IQR) box, with the whiskers representing the range. Isolates from the same participant and same ST have the fewest median SNPs. The Mann-Whitney U test was used to determinestatistical significance, where **** indicates P < 0.0001, and ns indicates no significances and P>0.05.

To determine the genomic diversity of longitudinal *S. aureus* isolates over time, SNP analyses were performed (Fig. 4 and **Table S5**). A median pairwise SNP distance of 2,344 (IQR 1178–38777) was observed for all the index (first isolate of a unique ST) isolates between participants. For all isolates between participants with the same ST, the median pairwise SNP difference dropped to 1,120 (IQR 614–1767), demonstrating that isolates from the same ST are more closely related to each other than to other STs (p-values < 0.0001), as expected^35^. Notably, within-participant longitudinal isolates, including those with ST changes, displayed a median pairwise SNP difference of eight (IQR 3–25). Furthermore, among within-participant longitudinal isolates of the same ST, the median pairwise SNP difference dropped to seven (IQR 2–13). Additional analyses of the longitudinal MRSA and MSSA populations from NHR 201 revealed the MSSA strains isolated across all five collection times differed by a median of 25 (IQR 21.75–29.75) SNPs (**Figure S5C**), while the median pairwise SNP difference among the MRSA strains isolated across the other 10 collection times was 67 (IQR 25.5–75.5) (**Figure S5D**). Together, these data demonstrate that within-participant longitudinally collected isolates are a highly genetically related, single lineage that persists over time despite repeated catheter exchanges and anti-staphylococcal antibiotic exposures.

### Antimicrobial susceptibility profiles of S. aureus LTUC isolates

**Figure 5:**
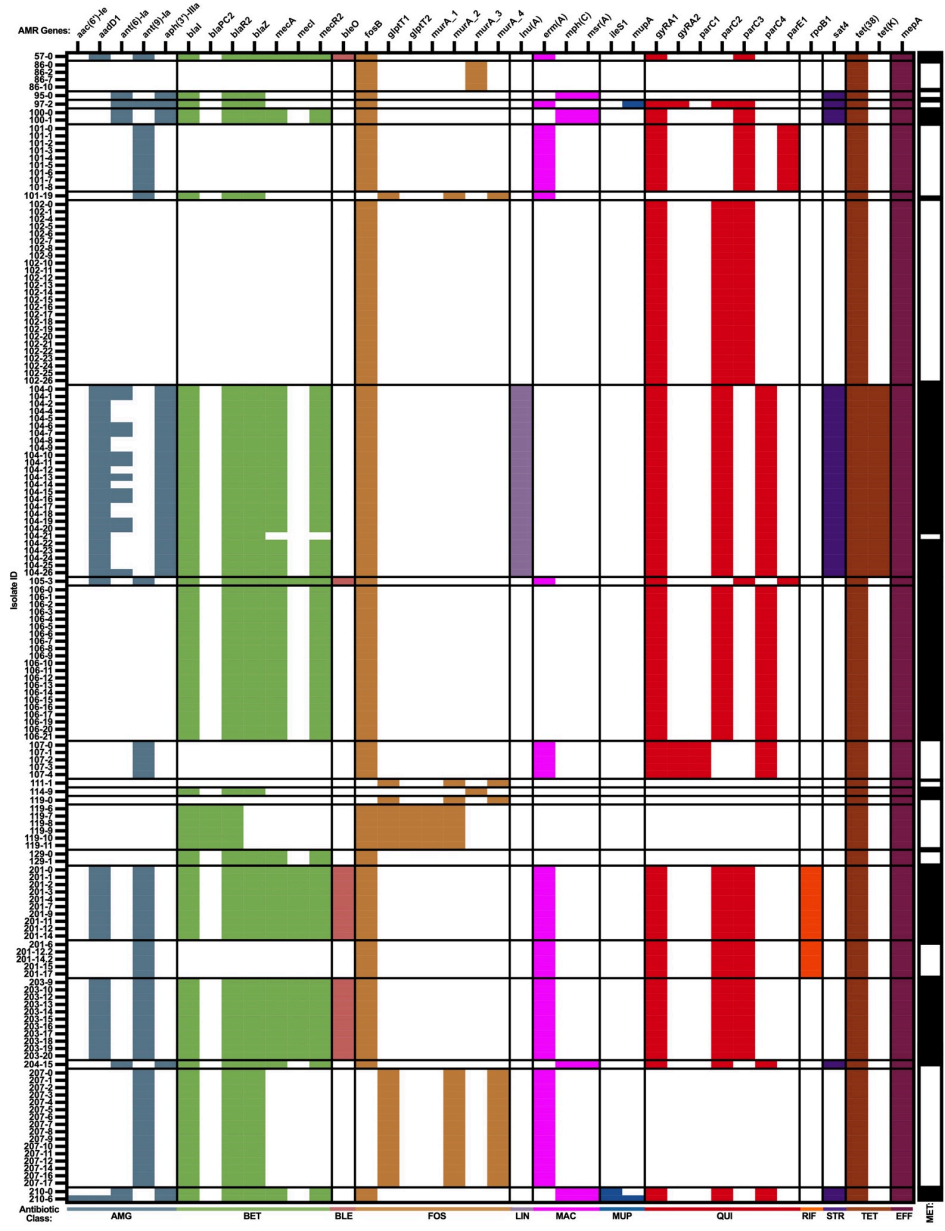
Antimicrobial resistance (AMR) gene carriage profile of *S. aureus* strains collected from individuals with long-term urinary catheters. *S. aureus*isolates are organized horizontally in chronological order with each strain labeled. The AMR genes are shown on top and are organized based on their associated antibiotic class. Antibiotic class is labeled below the head map and are organized in alphabetical order in which Aminoglycosides (AMG), Beta-Lactams (BET), Bleomycin (BLE), Fosfomycin (FOS), Lincosamide (LIN), Macrolide (MAC), Mupirocin (MUP), Quinolone (QUI), Rifamycin (RIF), Streprothricin (STR), and Tetracycline as (TE) based on AMRFinderPlus classification. Bars to the right of the graph indicate phenotypic methicillin resistance (black bars) and susceptibility (white bars) based on CSLI guidelines against oxacillin or cefoxitin. The following abbreviations were used glptT1 (glpT_A400V), glptT2 (glpT_V243I), murA_1 (murA_D278E), murA_2 (murA_E294D), murA_3 (murA_G257D), murA_4 (murA_T396N), ileS1 (ileS_V588F), gyRA1 (gyrA_S84L), gyRA2 (gyrA_S85P), parC1 (parC_E84G), parC2 (parC_E84K), parC3 (parC_S80F), parC4 (parC_S80Y), parE1 (parE_D432N), and rpoB1 (rpoB_H489N).

Most participants (12/20) received at least one antibiotic during the collection period (Fig. 1), and all isolates carried genes conferring resistance to multiple antibiotics (Fig. 5 and **Table S6**). Specifically, all isolates carried determinants that confer resistance to tetracyclines and Fosfomycin, and most encoded AMR genes associated with resistance to quinolones, macrolides, beta-lactams, and aminoglycosides. Only a few strains encoded AMR genes that confer resistance to lincosamide, rifamycin, mupirocin, bleomycin, and streptothricin. Importantly, the AMR determinant profiles were mostly stable among within-participant longitudinally collected isolates. Among the few isolates where AMR determinant instability was observed, these genes are associated with aminoglycoside resistance, which are generally carried on mobile genetic elements that are known to be readily gained and lost due to selective pressures. Additionally, a single instance of the loss of *mecA*-associated genes was observed in an isolate from NHR 104 (104 – 21). SNP analysis revealed that the previously persisting MRSA strain and the subsequent MSSA strain differed by only one SNP (a missense mutation in a hypothetical protein), indicating this strain lost the *mecA* cassette (**Table S6**).

Next, to determine the antimicrobial susceptibilities to the most commonly prescribed antibiotics for UTI, as well as some of the anti-staphylococcal antibiotics these participants received, minimum inhibitory concentration (MIC) assays were determined for index and final longitudinal isolates (Tables 2 and S7). Additionally, NHR 104 – 21 was included due to notable changes in its AMR determinant profile, specifically the loss of *mecA* when compared to the index and other longitudinal isolates from NHR 104 (Fig. 5). NHR 201 – 11 was also selected based on similar changes; *mecA* was absent in the final isolate (NHR 201 – 17) compared to the index isolate (NHR 201-0), and NHR 201 – 11 was the last longitudinal isolate encoding *mecA.* Antibiotics assessed included nitrofurantoin, trimethoprim-sulfamethoxazole, ciprofloxacin, oxacillin, penicillin, and vancomycin. All isolates assessed were susceptible to nitrofurantoin, trimethoprim-sulfamethoxazole, and vancomycin. Most index and final isolates (10/13) retained the same antibiotic susceptibility pattern. Phenotypic non-susceptibility to oxacillin was generally concordant with genotype, with strains encoding *mecA* displaying resistance to oxacillin, and those that did not, including those that lost *mecA* (e.g. NHR 104 – 21), displaying susceptibility. However, both strains isolated from UP 129 encoded *mecA,* yet the strains were phenotypically susceptible to oxacillin and are therefore classified as MSSA. A missense mutation in *mecA* (T547C) was observed in both isolates, potentially explaining the susceptibility. Furthermore, in contrast to the index isolate, UP 129’s final isolate was susceptible to penicillin, which may be due to an additional missense mutation (C299T) in *blaZ.* Together, these results demonstrate that there was concordance between genotypic and phenotypic antimicrobial susceptibility patterns of persistent *S. aureus* LTUC isolates, which remained largely stable despite exposures to antimicrobials.

### Carriage of AMR determinants and virulence factors is negatively correlated

**Figure 6:**
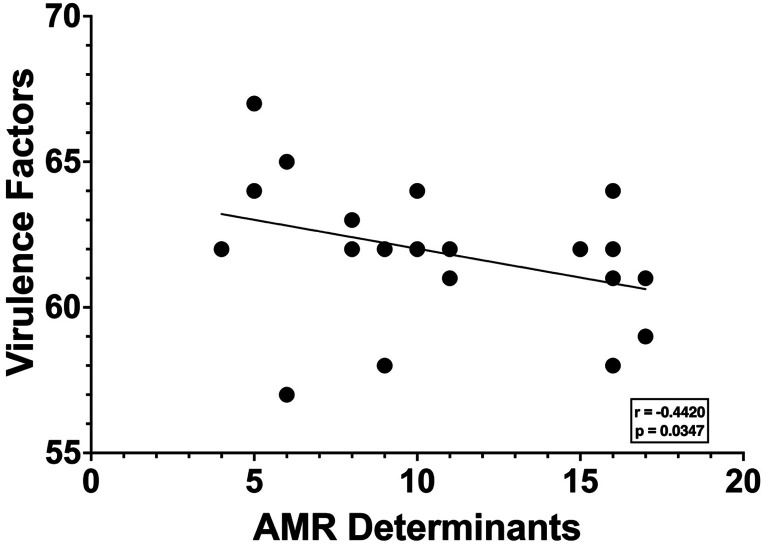
Correlation analysis between antimicrobial resistance (AMR) determinants and virulence genes. The total number of “resistance” determinants identified by AMRFinderplus were plotted against the total number of “virulence” genes identified by VFDB. A nonparametric Spearman’s correlation analysis was performed, and a significant correlation was found with p = 0.0347 and a r value of −0.442.

To determine the virulence factor carriage profile of *S. aureus* LTUC isolates, the virulence determinants encoded by all isolates were identified (**Figure S6 and Table S8**). Similar to the AMR determinants, virulence gene profiles remained stable for most longitudinal isolates. Additional correlation analyses of the index isolates were used to determine whether there is a relationship between the number AMR determinants and virulence genes encoded by uropathogenic *S. aureus.* A significant negative correlation was observed for the number of AMR determinants and virulence genes encoded (Fig. 6). Together, these data indicate that uropathogenic *S. aureus* strains that carry more AMR genes encoded fewer virulence factors.

## Discussion

Urinary catheterization is a common procedure that improves the quality of life for individuals with urinary tract dysfunction^4,5^. While this procedure is beneficial, urinary catheter duration is correlated with the risk of developing ASB and CAUTI, with longer dwell times associated with the highest risk^3,6-8,36^. Current treatment and prevention strategies include antimicrobial therapy and catheter exchanges^7,34^. Yet, recent studies demonstrate that prophylactic antibiotic administration does not prevent catheter colonization – even during short catheter durations – and may lead to more severe diseases, include *C. difficile* colitis, as well as the selection of AMR^3,11-14^. Additionally, CAUTIs continue to be one of the most common hospital-associated infections in the US^28,37,38^.

Previous studies indicate that the presence of *S. aureus* in the urinary tract *i)* is predominately associated with a urinary catheter, *ii)* is mainly MRSA, *iii)* can increase reflux to the kidney by blocking catheters via urease-produced encrustations, and *iv)* has a higher risk of disseminating to bacteremia compared to other uropathogens^9,12,29-33,39^. *S. aureus* is also a frequent cause of ASB, and persistent *S. aureus* ASB is associated with development of symptomatic infection^3,11,30^. Yet, *S. aureus* remains largely uncharacterized during its lifecycle in the catheterized urinary tract. This study analyzed longitudinally collected *S. aureus* strains isolated from two distinct cohorts: UPs and NHRs^3,11^. The demographics of both cohorts, including age, urinary catheter duration, and exposure to antibiotics, were similar. Whole-genome sequencing of all *S. aureus* strains revealed ST5 as the most frequently isolated lineage among both cohorts. However, the second most common ST differed between cohorts: ST8 and ST45 among UPs and NHRs, respectively. ST5, ST8, and ST45 are among the most frequently circulating lineages causing infection within the US^40-43^. Additionally, recent studies indicate ST5 is a common lineage found within the urinary tract globally^44,45^. While most mechanistic studies of *S. aureus* in the urinary tract assess ST8 isolates^29,45,46^, ST5 remains largely uncharacterized during CAUTI or ASB. Notably, the UP cohort was colonized with a wider diversity of STs compared to NHRs (eight vs four STs, respectively). This ST diversity may be due to the environmental differences within which UPs and NHRs reside^43,47,48^. NHRs live together in nursing homes, which may serve as a common environmental source of pathogen sharing. In contrast, UPs primarily reside at home within the community, resulting in potentially more diverse and participant-specific environmental sources of pathogen sharing.

*S. aureus* was present at the first collection time point for 65% (13/20) of participants across both cohorts. Importantly, among within-participant longitudinal isolates, the same ST was stably detected over time despite regular catheter changes (~ 27 days for UPs and ~ 38 days for NHRs). There were only two instances – one participant in each cohort (NHR 101 and UP 119) – where the index isolate ST changed to a different ST at subsequent collections. NHR 101 was initially colonized with ST5; however, after a catheter exchange and trimethoprim-sulfamethoxazole administration, *S. aureus* was no longer detected in the urine over the next 10 collections (10 weeks). At collection 19, an ST45 *S. aureus* was cultured from the urine. Similarly, the index isolate collected for UP 119 was ST59. *S. aureus* was not cultured from catheters over the next four collections (16 weeks), however, after ciprofloxacin treatment during collection four and five, an ST30 *S. aureus* was cultured from the catheter at collection six. This ST30 lineage persisted despite regular, monthly catheter exchanges until the end of the study period. Notably, among the 35% (7/20) of participants that acquired *S. aureus* during the collection period, all but one participant (NHR 204) received a catheter exchange or antibiotic exposure immediately prior to *S. aureus* isolation. Together, these data suggest that catheter exchanges and antibiotic exposures may disrupt communities, eliminating other uropathogens, leaving a niche open for *S. aureus* to rapidly recolonize. Additionally, while trimethoprim-sulfamethoxazole may have some efficacy at temporarily reducing *S. aureus* bacterial burdens in the catheterized urinary tract, this contrasts with other anti-staphylococcal antibiotics, such as nitrofurantoin^3,11,49^, which had no effect on the persistence of UP 86’s *S. aureus* isolates despite phenotypic susceptibility *in vitro.*

Phylogenetic analyses of longitudinal *S. aureus* strains demonstrate that within-participant isolates cluster more closely together compared to isolates from between participants, even among ST groups. Further genomic analyses indicated that within-participant isolates with the same ST were highly genetically related, with a median of four accessory genome changes and a median pairwise SNP difference of seven. For example, the 16 isolates from NHR 207 had a median of two accessory genome changes and a median pairwise SNP difference of seven. Additionally, among the four participants with co-isolates, genomic analyses demonstrated that these strains displayed a median of 2.5 accessory genome changes and a median pairwise SNP difference of five. Notably, while the MRSA and MSSA co-isolates from NHR 201 were from different lineages (72 accessory genome changes and SNP distance of 50) and the longitudinal MRSA strains were more heterogeneous (five accessory genome changes and SNP distance of 67), the longitudinal MSSA isolates were highly related to each other (three accessory genome changes and SNP distance of 25). NHR 201 had multiple antibiotic exposures over the course of the collection time, which may have provided additional pressures enabling more diverse lineage colonization. Interestingly, the MRSA strain colonized first, with the MSSA strain subsequently invading and eventually replacing the MRSA lineage. Taken together, these results are consistent with a previous study indicating a difference of 20 or fewer SNPs between strains can be used to identify a single, persisting *S. aureus* strain^50^. Additionally, these data demonstrate that *S. aureus* stably and asymptomatically persists within the catheterized bladder for up to nine months, resulting in persistent ASB. Importantly, most participants are dominated by a single homogeneous population that is highly genetically related, indicating a single lineage persists over time. This pattern was observed across geographically distinct sites (Midwest vs Northeast), suggesting these results are broadly applicable to individuals with LTUCs colonized with *S. aureus.* Impactfully, the observed SNP increases are greater than the expected average accumulation of 2.72 SNPs per megabase per year observed when *S. aureus* stably colonizes the nares^51^, indicating *S. aureus* may be adapting to the urinary tract or exposure to antibiotics over time.

Consistent with previous reports^9,30^, half of the *S. aureus* LTUC strains carried *mecA* and were resistant to oxacillin, making them MRSA. Additionally, most LTUC strains carry genes that confer resistance to a broad array of antibiotic classes, including fosfomycin and tetracyclines. Importantly, AMR gene carriage was stable across longitudinal isolates, despite most participants receiving at least one antibiotic during the study period and many receiving anti-staphylococcal antibiotics, including ciprofloxacin (5/20), trimethoprim-sulfamethoxazole (3/20), nitrofurantoin (3/20), and cephalexin (2/20)^3,11^. MIC assays using the antibiotics participants received or those that are commonly used to treat CAUTI demonstrated that antibiotic susceptibility patterns were stable among longitudinal isolates, with most strains (both index and final) displaying resistance to ciprofloxacin but susceptibility to nitrofurantoin. Despite treatment with antibiotics to which these isolates should be susceptible, the same strain was found to persist over the course of 12 months. For example, UP 86 received ciprofloxacin and nitrofurantoin over the course of the collection period^11^ and the index and final isolates (collected 12 months apart) were intermediate resistant and susceptible, respectively.

Of the few strains that exhibited discordant antimicrobial susceptibility patterns between the index and final isolates, the most notable were the NHR 201 MRSA and MSSA isolates. However, the NHR 201 index and final MRSA isolates and the final MSSA isolate exhibited concordant genotypic and phenotypic beta-lactam susceptibility, with the differences due to invasion and replacement by a different lineage. Additionally, MRSA stably colonized NHR 104 for 26 weeks, yet *mecA* was lost at a single collection time (21), making it a MSSA strain. Since loss of *mecA* is rare, additional analyses performed determined that the MSSA and MRSA strains were the same ST and the strains collected at time point 0 (MRSA) and 21 (MSSA) differed by only one SNP, which occurred in a hypothetical protein. These results demonstrate that the MSSA and MRSA are the same strain, and that, while rare, *mecA,* and therefore resistance to beta-lactams, was lost during persistent *S. aureus* ASB. In contrast to the index isolate, UP 129’s final isolate was also susceptible to penicillin likely due to a missense mutation (C299T) in *blaZ,* a gene that encodes a beta-lactamase. MIC assays with oxacillin confirmed the predicted genotypic susceptibility of longitudinal isolates was generally concordant with phenotypic susceptibility. The only exceptions to this concordance were isolates from UP 129, which encoded the Scc *mec* Type IVc(2B), yet were phenotypically susceptible to oxacillin and therefore MSSA. A missense mutation in *mecA* (T547C) was observed in both isolates, potentially explaining the susceptibility. Together, these results highlight that LTUC isolates are multidrug resistant, further strengthening the hypothesis that ASB serves as a reservoir for drug resistant *S. aureus.* In addition to AMR genes, virulence factor carriage was also stable among longitudinal isolates. Impactfully, carriage of AMR genes was inversely correlated with the presence of virulence factors: strains with increased AMR determinants encoded fewer virulence factors. Notably, the stable carriage of AMR genes across longitudinal *S. aureus* LTUC isolates may help guide the selection of empiric antibiotics for treatment of subsequent CAUTI and therefore improve patient outcomes. Challengingly, these data also suggest that phenotypic resistance does not always correlate with *S. aureus* persistence, as some strains that were susceptible to the antibiotics participants received during the collection period persisted as ASB. Thus, studies are needed to further dissect the *S. aureus* recalcitrance mechanism that promote persistence.

This study was limited by the scope of both cohorts, which prioritized the isolation and sequencing of a single isolate per species for each participant at each collection. This collection strategy may limit the detection of heterogenous populations that may be present, as shown with NHR 201. Additionally, only the NHR cohort was designed to differentiate symptomatic CAUTI from ASB, which limited our ability to determine if strains persisting as ASB transitioned to cause symptomatic CAUTI in our UP cohort. Finally, while we used both long-and short-read sequencing for index isolates, only short-read sequencing was performed for longitudinal- and co-isolates. This approach allowed us to define the genomic synteny (long reads) and polish the genomes (short reads) of the index isolates to assess SNPs over time. However, without long-reads for the longitudinal and co-isolates, we may have missed large scale structural variations or gene gain/loss events that resulted from the movement or acquisition of mobile genetic elements. Notably, however, we were able to identify longitudinal and co-isolates with large accessory genome changes, limiting this concern. In depth analyses of these observations indicated two likely causes: 1) ST switches within participants suggest these changes were due to strain lineage differences, and 2) loss of large mobile genetic elements, including the *mecA* cassette, with few or no SNPs differences, indicate these are the same strain with gene loss. Nevertheless, most longitudinal and co-isolates were highly genetically related, and the pan-genome repertoire of each longitudinal set of isolates was stable, suggesting there are minimal longitudinal structural differences within most of the strain collection. Thus, most strains stably persist as ASB during long-term catheterization.

Our study demonstrates that a single *S. aureus* strain persists over time despite antibiotic exposure or catheter exchanges, which are the two accepted practices for reducing microbial burdens during catheterization^7,34^. Importantly, these results are broadly applicable to LTUCs colonized with *S. aureus,* as this pattern was observed across geographically distinct sites. Furthermore, while all strains tested are susceptible to nitrofurantoin, most strains (> 61%) are resistant to ciprofloxacin, which are two commonly prescribed antibiotics for the treatment of symptomatic CAUTI^7,11,28,52^. The ability of *S. aureusto* persist despite susceptibility to the antibiotics participants received during the collection period warrants further investigation into the mechanisms driving this phenotypic recalcitrance. Importantly, the stable carriage of AMR determinants among longitudinal isolates suggests that monitoring AMR patterns of persistent *S. aureus* ASB isolates may help guide physician selection of antibiotics to treat subsequent symptomatic CAUTI, which may improve the efficacy of empiric therapy and therefore patient outcomes. Finally, the accumulation of SNPs within longitudinal isolates suggest uropathogenic *S. aureus* LTUC isolates are under selection. Future studies are needed to determine if and how *S. aureus* is adapting to selective pressures, such as the urinary tract or antibiotics.

## Methods

### Strains and growth conditions.

All strains used in this study are from individuals with LTUCs from two separate, previously published cohorts and are listed in (**Table S1**)^3,11,12^. The first cohort is comprised of individuals seen at the Division of Urology at Washington University School of Medicine in St. Louis in Missouri^11,12^. Isolates were collected from rich media based on unique colony morphology and 16S sequencing confirmed the species. A total of 38 *S. aureus* strains were collected from 2018–2021, deidentified, and used as a part of this study. The second cohort is comprised of individuals residing in two separate nursing home facilities in New York^3^. Isolates were collected from selective media and differentiated by colony morphology with additional testing performed to identify species. A total of 133 *S. aureus* strains were collected from 2019–2020, deidentified, and used in this study. Mannitol Salt Agar (BD, cat 211407) plates were used to confirm and isolate all *S. aureus* clinical isolates from both cohorts prior to whole-genome sequencing. Brain-heart infusion (BHI) broth (BD, cat. 237200) and agar (BD, cat. 214010) were used to maintain strains. Tryptic Soy Broth (TSB) (Sigma, cat. #22092-500G) was used to grow isolates for whole-genome sequencing. For MIC assays, bacterial isolates were grown in Mueller-Hinton broth (MHB) (Sigma; cat. 7019-100G) and MHB supplemented with 2% NaCl for oxacillin, according to the Clinical Laboratory Standard Institute (CLSI)^53^. Methicillin resistance using cefoxitin was previously reported for the NHR cohort^3^. Oxacillin resistance was confirmed for index and final isolates as described below.

### Whole-genome sequencing of the S. aureus isolates.

Whole-genome sequencing of all isolates (**Table S1**) was performed as previously described^54^. Briefly, *S. aureus* strains were grown in TSB for 2–4 h, shaking at 37°C. The cultures were pelleted and genomic DNA was extracted using the Qiagen QIAamp DNA minikit (Qiagen, cat. 51306) following the manufacturer's protocol, with the exception that 1 μg/mL of lysostaphin and 20 μg/mL of lysozyme was added to the initial buffer. Genomic DNA then underwent library preparation for short-read sequencing using the Illumina NextSeq 2000 sequencer. Genomes from short reads were assembled as previously described^54^. Briefly, read quality assessment was conducted with Raspberry v0.3, trimming was conducted with Trimmomatic v0.39, SPAdes v3.15.2 was used for assembly, and assembly quality was assessed using Quast v5.2.0^54^. Additional assembly quality control measures include BUSCO v5.7.0, for genome completeness, and CheckM v1.1.2, for genome quality control (**Tables S2-3**)^55,56^. Isolates were re-sequenced if the genome had > 500 contigs, had a genome size greater or less than 2 standard deviations from the average of all *S. aureus* genomes within NCBI, or if the assembly was contaminated with another bacterial species as defined by the presence of another bacterial species identified at greater than 2% by StrainSeeker v1.5^57^. Additionally, a subset of isolates was selected for Oxford Nanopore long-read sequencing to accurately reconstruct genome structures, including the circularization of plasmids and resolution of mobile genetic element integration. Isolates were chosen based on the identified ST from short-read sequencing, with the first isolate with a unique ST within a participant’s longitudinal set selected for long-read sequencing, which we define as an index isolate (**Table S1**). Briefly, the Oxford SQK-RBK004 library preparation kit was used to prepare bacterial DNA for sequencing on the Oxford Nanopore GridION X5 (Oxford, UK) using FLO-MIN106 flow cells. Genomes were assembled with Flye v2.9.1 and then underwent error correction and polishing steps using Racon v1.5.0 and Medaka to generate hybrid assemblies as previously described^58^.

### Molecular epidemiology of the bacterial assemblies

MLST v2.19.0 was used to identify the ST for each isolate as previously described^54,59^. Gene annotations for each assembled genome were performed via Prokka v1.14.5 as previously described^54,60^.

### Pan-genome and phylogenetic analysis of the S. aureus isolates

The core genome for all isolates in the dataset, including index, co-isolates, and each set of longitudinal isolates for a participant, was identified with Roary v3.13.0 on the annotated short-read assemblies^61^. Accessory gene lists obtained from the Roary output were defined as genes present in > 99% of isolates. Additionally, to quantify the gene gain/loss events for each set of longitudinal and co-isolates from a participant, the accessory genome changes were identified by comparing the gene content of the index isolate to the longitudinal or co-isolated strains. Participants with single isolates were not included as no changes in gene content could be quantified. Final analysis and visualization were made in GraphPad Prism version 10.1.1. The core-gene alignment file from Roary was used with RAxML v8.2.12 to produce maximum-likelihood phylogenetic trees that were visualized using iTOL and edited on Affinity Designer as previously described^62-64^.

### SNPs analyses of clinical isolates

SNP analyses were conducted using Snippy v4.6.0, aligning the paired-end reads from the queried isolate to the index isolate (**Table S9**)^65^. For co-isolated strains, short-read assembled genomes were used to compare the index sequence to the sequences of the other co-isolated strains. To reduce potential bias in our longitudinal analyses, co-isolated strains of the same ST and *mecA* carriage as the index isolate were excluded as they had an average of less than 5 SNPs. In instances where two or more isolates were collected during the index collection, catheter isolates were prioritized. To identify genomic differences in our longitudinal cohort, the hybrid assembly of the index isolate from each set, defined as the first isolate of an ST within a participant, was used as a reference for each longitudinal strain to enable comprehensive assessment of whole-genome SNPs, including those in intergenic regions, and resolve the genomic synteny of these mutations. Self-self comparisons (i.e. comparing the index isolate’s short reads against the hybrid assembly) were excluded from statistical assessments as short-reads were used to polish the long-read assemblies to remove errors. Final analysis and visualization were made in GraphPad Prism version 10.1.1.

### Antimicrobial resistance and virulence factor profiles

The AMR profile of each strain was assessed using NCBI AMR Gene Finder Plus (AMRFinderPlus v3.11.8) for both mutational and acquired AMR mechanisms as well as species-specific virulence determinants (**Tables S5-7**), as previously described^66,67^. Virulence factors were obtained using Abricate (https://github.com/tseemann/abricate) using the virulence factor database (VFDB), as previously described^58,68^. This data set was prioritized for analyses as it encompassed a larger repertoire of determinants compared to AMRFinder Plus virulence determinants (**Table S8**). Additionally, the *mecA* cassette type was identified using the Scc*mec* Finder 1.2 webtool^69^. The number of AMR determinants identified within the index isolate was compared to the number of virulence genes and a nonparametric Spearman’s correlation analysis was used to determine statistical correlation. Longitudinal isolates were excluded as most AMR and virulence profiles remained consistent, thus including them would violate the assumption of independence required for the statistical test.

### Minimum inhibitory concentration assays

MICs were determined based on the 2024 CLSI guidelines as previously described^58^. Briefly, isolates were grown to ~ 2.8E8 colony forming units (CFU)/mL in MHB or MHB supplemented with 2% NaCl for oxacillin per the CLSI guidelines, corresponding to an optical density at 600nm (OD_600_) of 0.4 using a Synergy H1 Biotek microtiter plate reader^53^. Cultures were then diluted to ~1E6 CFU/mL and equal volumes of antibiotic and bacteria were transferred to a 96-well plate with final concentrations corresponding to the sensitive or resistant MIC breakpoints of each antibiotic tested and ~ 5E5 CFU/mL. The final antibiotic concentrations used were as follows: 1, 2, and 4 μg/ml ciprofloxacin, 32 and 128 μg/ml nitrofurantoin, 2 and 4 μg/ml oxacillin, 0.125 and 0.25 μg/ml penicillin, 2/38 and 4/76 μg/ml trimethoprim-sulfamethoxine, and 1 and 16 μg/ml vancomycin. Plates were incubated at 37° or 35°C overnight following CLSI incubation times. Resistance was determined based on an OD_600_ of greater than 0.1 and each condition consisted of at least 4 replicates – 2 technical replicates and 2 biological replicates (**Table S9**).

### Statistical Analysis

Statistical significance between epidemiological descriptions was determined using the Fisher’s exact and the Mann-Whitney test. Significant differences for SNPs comparisons were determined using the Mann-Whitney U test. The Pearson’s correlation analysis was used to determine the correlation between number of antimicrobial resistance and virulence factors. All statistical tests were performed using GraphPad Prism version 10.1.1.

## Figures and Tables

**Table 1:** Participant demographics and epidemiology of clinical isolates from participants with *S. aureus* colonization of long-term urinary catheters. Statistical analyses were performed using the Fisher’s exact test denoted with * or the Mann-Whitney test denoted with ^#^.

	Urology Outpatients (UP)	Nursing Home Resident (NHR)	p-value
Participants with *S. aureus*	10	10	NA
Female Participants	50%	20%	0.3498*
Percent of Participants with MRSA Colonization	40%	60%	0.6563*
Average Antibiotic Treatments per participant	2.9	1.5	0.2867#
Average Catheterization Duration	27.40 days	38.12 days	0.6698#
Number of *S. aureus* Isolates	38	133	NA
Average Number of *S. aureus* isolates per participant	3.8	13.3	NA

**Table 2.** Minimum inhibitory concentration (MIC) assays assessing susceptibilily of longitudinally collected *S. aureus* isolates to commonly used antibiotics and antibiotics participants received during the collection time – nitrofurantoin (Nitno) ciprofloxacin (Cipro), oxacillin (Oxa), penicillin (Pen), vancomycin (VanC) and trimethoprim-sulfamethoxazole (TMP/Sulfa). S = susceptible, I = intermediate, R = resistant, black boxes = not assessed.

Isolate ID	Nitro	Cipro	Oxa	Pen	Vanc	TMP/Sulfa
**86-0**	S	I	S	S		
**86-10**	S	I	S	S		
**100-0**	S	R	R	R		
**100-1**	S	R	R	R		
**101-0**	S	R	S	S	S	
**101-8**	S	R	S	S	S	
**102-0**	S	R	S	S		
**102-26**	S	R	S	S		
**104-0**	S	R	R	R		
**104-21**	S	R	S	R		
**104-26**	S	R	R	R		
**106-0**	S	R	R	R		
**106-21**	S	R	R	R		
**107-0**	S	S	S	S		
**107-4**	S	S	S	S		
**119-6**	S	S	S	R		
**119-11**	S	S	S	R		
**129-0**	S	S	S	R		
**129-1**	S	S	S	S		
**201-0**	S	R	R	R	S	S
**201-11**	S	R	R	R	S	S
**201-17**	S	R	S	S	S	S
**203-9**	S	R	R	R		
**203-20**	S	R	R	R		
**207-0**	S	S	S	R		
**207-17**	S	S	S	R		
**210-0**	S	R	S	R		S
**210-6**	S	R	S	R		S

## Data Availability

Annotated genomes were deposited into NCBI and are available under the BioProject ID: PRJNA1265249.
